# Correction to “Dermoscopic Features Associated with 3‐GEP PLA: LINC00518, PRAME, and TERT Expression in Suspicious Pigmented Lesions”

**DOI:** 10.1111/srt.70305

**Published:** 2025-12-16

Ludzik J, Becker AL, Latour E, Lee C, Witkowski A. Dermoscopic features associated with 3‐GEPPLA: LINC00518, PRAME, and TERT expression in suspicious pigmented lesions. *Skin Res Technol*.2023;29:4:e13323. doi: 10.1111/srt.13323

In the published version, the institutional affiliation of author Joanna Łudzik was incomplete. Only the Oregon Health & Science University affiliation was listed. The correct affiliations should read: Joanna Łudzik, Oregon Health & Science University, Department of Dermatology, Portland, USA; Jagiellonian University Medical College, Department of Bioinformatics and Telemedicine, Kraków, Poland.

We apologize for this error.

